# Local Trends in Total Joint Arthroplasty and Orthopaedic Surgeon Distribution in the United States

**DOI:** 10.5435/JAAOSGlobal-D-22-00114

**Published:** 2022-07-06

**Authors:** Christopher M. Scanlon, Brian A. Perez, Austin Yu, Matthew Sloan, Amanda Milena Alvarez, Matthew L. Webb, Neil P. Sheth

**Affiliations:** From the Department of Orthopaedic Surgery, Hospital of the University of Pennsylvania (Dr. Scanlon, Dr. Perez, Dr. Sloan, Dr. Webb, and Dr. Sheth); the Drexel University College of Medicine (Dr. Yu); and the Department of Political Science, Temple University, Philadelphia, PA (Dr. Alvarez).

## Abstract

**Introduction:**

Total joint arthroplasty (TJA) volume and the number of orthopaedic surgeons in the United States have increased in recent years, but local growth variation has not been studied. This study assesses recent changes in state-level distribution of orthopaedic surgeons in the United States and corresponding local trends in TJA volume.

**Methods:**

Data from the National Inpatient Sample database (2000 to 2014) were reviewed. Urban versus rural setting and teaching versus nonteaching hospitals were identified among TJA procedures for comparison. Data from the American Academy of Orthopaedic Surgeons (2002 to 2016) detailing orthopaedic surgeon practice location were evaluated, and linear regression analysis was used to correlate state population data with orthopaedic surgeon density.

**Results:**

From 2000 to 2014, there was a 0.1% to 0.3% (*P* < 0.01) annual decrease in the proportion of TJA procedures conducted in rural hospitals. No notable change was observed in the proportion of TJA procedures conducted at urban teaching versus nonteaching hospitals. Linear regression analysis demonstrated that decreased state population was associated with higher orthopaedic surgeon density (adjusted R^2^ = 0.114, *P* < 0.01). States with a higher percentage of population living in rural areas had a lower density of orthopaedic surgeons in the South region and a higher density of orthopaedic surgeons in the remainder of the county.

**Conclusions:**

Less populated, rural states have a higher density of orthopaedic surgeons than states with increased population and less rural areas. Although TJA volume has increased since 2000, the proportion of TJA procedures conducted at rural hospitals has decreased. No change was found in the proportion of TJA procedures conducted at urban teaching versus nonteaching hospitals. This may indicate that more patients living in rural areas are seeking TJA care in urban centers. Future studies are needed to confirm this and ensure that patients living in rural areas have appropriate access to TJA care.

Total joint arthroplasty (TJA) volume in the United States (US) has continued to increase over the past 20 to 30 years^1,2^ and is expected to rise steeply over the next decade.^3-5^ Efforts to address this demand have resulted in considerable growth in the population of practicing orthopaedic surgeons since the turn of the century.^6,7^ A recent analysis from our institution demonstrated that these efforts have resulted in a 51% increase in the number of orthopaedic surgeons per capita since the turn of the century and projected a continued per capita growth rate of 40% over the next decade.^8^ However, because the orthopaedic surgeon workforce continues to age as a whole,^9^ there are persistent concerns about the ability of the workforce to address the increasing demand for orthopaedic services,^3^ especially about demand for TJA procedures. This is particularly true for the South region, which was shown in our study to be at an increased risk of experiencing a shortage of orthopaedic surgeons over the next decade as compared with the rest of the country.^8^

Although numerous studies have assessed the recent increase in TJA volume and have projected future growth,^1,2,3,4,5,9^ to the best of our knowledge, no studies have evaluated trends at the local level. In addition, although we have recently evaluated regional variations in orthopaedic surgeon density and growth,^8^ orthopaedic surgeon density at the state level has not been evaluated. Understanding both local trends in TJA volume and the state-level distribution of orthopaedic surgeons is vital to identify mechanisms to better distribute the burden of increased demand for TJA procedures over the next decade.

To better understand the settings in which patients receive TJA services at a local level, we evaluated practice trends regarding the types of hospitals (i.e., rural versus urban and teaching versus nonteaching hospital) where TJA procedures were conducted since 2000. We concurrently assessed recent trends in orthopaedic surgeon distribution at the state level and compared orthopaedic surgeon density with overall state population and population density; a particular emphasis was placed on the South region as compared with the rest of the country because of the findings of our recent study.^8^ We hypothesized that TJA volume in both rural and urban settings as well as teaching and nonteaching hospitals would consistently increase over the study period. We further hypothesized that orthopaedic surgeon density at the state level would vary with state population and that states with low population densities and large rural areas would have a lower density of orthopaedic surgeons.

## Methods

### Data Extraction

A retrospective review of data from the American Academy of Orthopaedic Surgeons (AAOS) and the National Inpatient Sample (NIS) database was conducted. The NIS database was queried for patients who had undergone primary or revision THA or TKA between 2000 and 2014. The NIS database contains a representative national sample of all discharges among hospitals in the United States in a calendar year. Over 116 million records were included for analysis. Patients undergoing primary or revision TJA were identified by the International Classification of Disease, Ninth Edition, procedure code (81.51 for THA, 81.54 for TKA, 81.53, 00.70 to 00.73 for revision THA, and 81.55, 00.80 to 00.84 for revision TKA). The total number of patients who had undergone any of these procedures was extracted for each year during the study period. The location of the conducted procedure (urban versus rural setting) and whether urban setting procedures were conducted in a teaching versus nonteaching hospital were extracted from the database. These hospital designations were provided for each procedure in the NIS database.

Information on the number of practicing orthopaedic surgeons in the United States was obtained from the biennial Orthopaedic Practice in the United States report published by the AAOS.^7,10,11,12,13,14,15,16^ The total number of orthopaedic surgeons practicing in the United States, as well as the total number of orthopaedic surgeons per state based on the practice location from 2002 to 2016, was obtained from these reports. In addition, information on state population, population density, and percentage of state population living in rural areas for each of the 50 states and Washington D.C. was obtained from the US Census Bureau. Rural and urban areas were delineated using the definition put forth by the US Census Bureau, which is complex but defines urban areas as those that have a population density of 1000 people per square mile and defines rural areas as any area that is not urban.^17^

### Data Analysis

The number of patients who underwent TJA in a rural or urban setting and in a teaching hospital or a nonteaching hospital was extracted from the NIS and compared by year. Chi square analysis was conducted to determine trends in utilization rates of urban versus rural hospital setting and teaching versus nonteaching hospitals among urban hospitals over the study period among the first and last study years. In addition, the number of TJA procedures conducted in urban versus rural areas per capita was calculated for the study period based on NIS data and data from the US Census Bureau.^18^ The z-statistic was calculated for population-level data, to compare changes in per capita TJA volume over the study period.

The number of practicing orthopaedic surgeons per capita in the United States and in each state based on the practice location was determined based on published AAOS data and US Census Bureau data.^18^ State-level data for orthopaedic surgeon density were obtained from the Orthopaedic Practice in the United States 2016 report from the AAOS,^7^ and state-level population data for each state and the District of Columbia (D.C.) in 2016 were obtained from US Census Bureau data.^18^ Linear regression was conducted to compare orthopaedic surgeon density with overall state population and state population density to evaluate for trends in the number of orthopaedic surgeons per capita compared with overall population at the state level. This was repeated for only those states in the South region (as defined by the US Census Bureau^19^; Table 1) and for the remainder of the states excluding those states in the South region (not south, NS). The South region was of particular interest because of previous work demonstrating this region to be at an increased risk of experiencing a shortage of orthopaedic surgeons in the coming decade.^8^ We therefore wanted to evaluate trends in the South region as compared with the rest of the country to help identify strategies to ameliorate this risk.

**Table 1:** List of States in Each of the Unites States

Region	States
Northeast	Connecticut, Maine, Massachusetts, New Jersey, New Hampshire, New York, Pennsylvania, Rhode Island, and Vermont
Midwest	Illinois, Indiana, Iowa, Kansas, Minnesota, Michigan, Missouri, Nebraska, North Dakota, Ohio, South Dakota, and Wisconsin
South	Alabama, Arkansas, Delaware, Florida, Georgia, Kentucky, Louisiana, Maryland, Mississippi, North Carolina, Oklahoma, South Carolina, Tennessee, Texas, Virginia, and West Virginia
West	Alaska, Arizona, California, Colorado, Hawaii, Idaho, Montana, Nevada, New Mexico, Oregon, Utah, Washington, and Wyoming

Census Bureau–designated statistical regions.

States in the South region were included in the South region group, and all other states were included in the Not-South (NS) group for comparison.

In addition, linear regression analysis was conducted to compare the orthopaedic surgeon density of all states with the percentage of the population living in rural areas in each state to determine the association between states' rural population and orthopaedic surgeon supply. This was repeated for the South region and the NS group. Stata Statistical Software, version 14.2 (StataCorp) was used for all statistical analyses.^20^

## Results

### Local Trends in Total Joint Arthroplasty Volume

Using the NIS data set, which is a sample of the number of TJAs conducted in the country, we were able to extrapolate the total number in our analysis. From 2000 to 2014, TJA volume in the United States increased from 494,005 procedures to 1,166,121 procedures annually (136% increase) (Table 2). Over this period, the number of TJA procedures conducted in rural areas increased by 54.3%. A small but statistically significant decrease (0.1% to 0.3% per year, *P* < 0.01) was observed in the proportion of TJA procedures that were conducted in hospitals located in a rural area over the study period, such that 13.7% of all TJA procedures were conducted in rural hospitals in 2000 while only 8.9% of TJA procedures were conducted in rural hospitals by 2014.

**Table 2:** Comparison of the Number of TJA Procedures Conducted in Urban and Rural Hospitals Over the Course of the Study Period

Year	Urban TJA Procedures	Rural TJA Procedures	Total TJA Procedures
2000	426,170 (86.3%)	67,835 (13.7%)	494,005
2014	1,061,358 (91.1%)	104,763 (8.9%)	1,166,121

TJA = total joint arthroplasty

The total number of procedures conducted in urban and rural hospitals is reported for the year 2000 and 2014. The percentage of total number of TJA procedures conducted is in parentheses. Data were obtained from the National Inpatient Sample database, which includes designation of whether the hospital where the procedure was conducted is located in an urban or rural area.

Among hospitals located in an urban area, total TJA volume increased 149% from 2000 to 2014. No notable change was observed in the proportion of TJA procedures conducted at urban teaching hospitals as compared with urban nonteaching hospitals over the study period.

Similar trends were seen when evaluating the number of TJA procedures that were conducted on a per capita basis in urban and rural areas from 2000 to 2014 (Table 3). In 2000, there was an increased number (*P* < 0.001) of TJA procedures conducted per capita in urban areas (1.92 per 1000 residents) as compared with rural areas (1.15 per 1000 residents). Over the study period, urban TJA volume per capita increased at a significantly (*P* < 0.001) higher rate (115.5%) than rural TJA volume per capita (48.4%). This resulted in a substantial increase in the discrepancy between the number of TJA procedures conducted in urban areas as compared with rural areas over the study period because urban TJA volume per capita was 66.9% higher than rural TJA volume per capita in 2000 and 142.3% higher in 2014.

**Table 3:** Comparison of the Number of TJA Procedures Conducted per 1000 Persons in Urban and Rural Areas in the United States From the Year 2000 to 2014

Year	2000	2014	Percentage Increase
Urban TJA procedures (per 1000 persons)	1.92	4.13	116.5
Rural TJA procedures (per 1000 persons)	1.15	1.71	48.4
Total TJA procedures (per 1000 persons)	1.76	3.66	109.6

TJA = total joint arthroplasty

The number of TJA procedures is reported per 1000 persons residing in urban and rural areas, as well as the number of TJA procedures conducted per 1000 persons in the United States. Data were obtained from the National Inpatient Sample database, which includes designation of whether the hospital where the procedure was conducted is located in an urban or rural area. Urban, rural, and overall US population data were obtained from the US Census Bureau.

### Orthopaedic Surgeon State-level Distribution Trends

**Figure 1:**
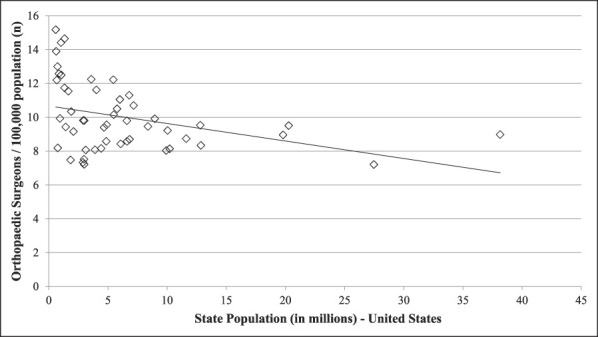
Graph showing linear regression analysis for orthopaedic surgeon density as a function of state population. There was a trend toward decreased orthopaedic surgeon density with an increase in overall state population (adjusted R^2^ = 0.114, *P* < 0.01). Orthopaedic surgeon density was obtained from the Orthopaedic Practice in the United States 2016 report from the AAOS and is reported as the number of orthopaedic surgeons per 100,000 residents. State population information was obtained from the US Census Bureau and is reported in millions. AAOS = American Academy of Orthopaedic Surgeons

**Figure 2:**
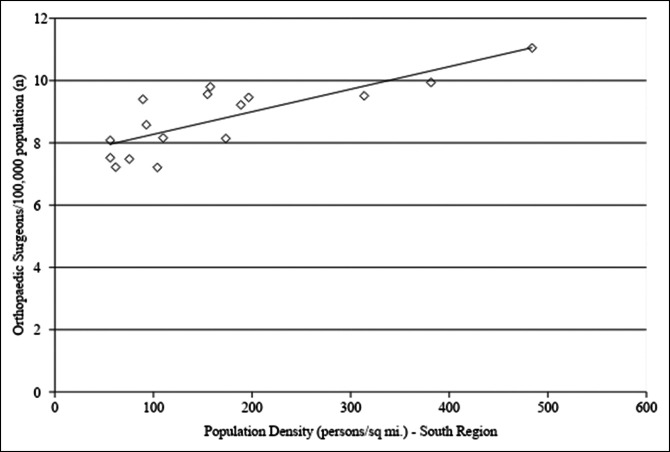
Graph showing linear regression analysis for orthopaedic surgeon density as a function of state population density for the South region. There was a trend toward increased orthopaedic surgeon density with an increase in state population density (adjusted R^2^ = 0.609, *P*=<0.01). Orthopaedic surgeon density was obtained from the Orthopaedic Practice in the United States 2016 report from the AAOS and is reported as the number of orthopaedic surgeons per 100,000 residents. State population density information was obtained from the US Census Bureau and is reported in number of persons per square mile. AAOS = American Academy of Orthopaedic Surgeons

Linear regression analysis of orthopaedic surgeon density related to overall population at the state level demonstrated that decreased state population was associated with higher orthopaedic surgeon density (adjusted R^2^ = 0.129, *P* < 0.01, Figure 1). No association was observed between orthopaedic surgeon density and state population density when evaluating all states (*P* = 0.259) and the NS group (*P* = 0.931). However, there was a significant positive association between orthopaedic surgeon density and state population density in the South region (adjusted R^2^ = 0.609, *P*=<0.01, Figure 2).

**Figure 3:**
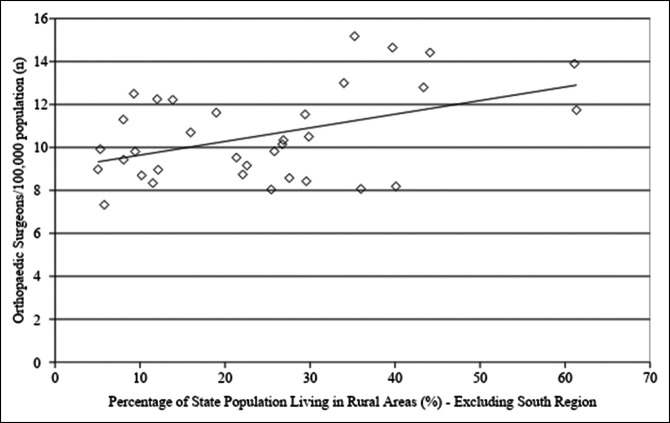
Graph showing linear regression analysis for orthopaedic surgeon density as a function of the percentage of states' population living in rural areas in all states excluding the South region (NS group). There was a trend toward increased orthopaedic surgeon density with an increase in the percentage of state population living in rural areas (adjusted R^2^ = 0.175, *P* = 0.008). Orthopaedic surgeon density was obtained from the Orthopaedic Practice in the United States 2016 report from the AAOS and is reported as the number of orthopaedic surgeons per 100,000 residents. Information on the percentage of states' populations living in rural areas was obtained from the US Census Bureau and is reported as a percentage. AAOS = American Academy of Orthopaedic Surgeons

**Figure 4:**
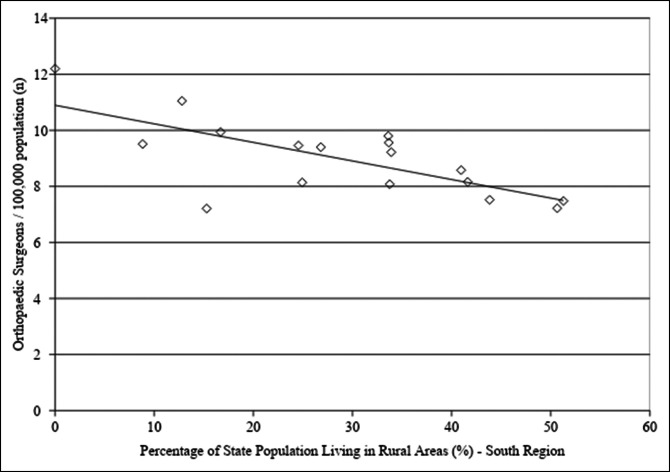
Graph showing linear regression analysis for orthopaedic surgeon density as a function of the percentage of states' population living in rural areas in states in the South region. There was a trend toward decreased orthopaedic surgeon density with an increase in the percentage of state population living in rural areas (adjusted R^2^ = 0.469, *P* < 0.01). Orthopaedic surgeon density was obtained from the Orthopaedic Practice in the United States 2016 report from the AAOS and is reported as the number of orthopaedic surgeons per 100,000 residents. Information on the percentage of states' populations living in rural areas was obtained from the US Census Bureau and is reported as a percentage. AAOS = American Academy of Orthopaedic Surgeons

Linear regression analysis was conducted to evaluate the association between states' rural population and orthopaedic surgeon density. When evaluating all states, there was no association between rural population percentage and orthopaedic surgeon density (*P* = 0.478). However, when evaluating the NS group, there was a positive correlation between the two, such that an increased state rural population percentage was associated with increased orthopaedic surgeon density (adjusted R^2^ = 0.175, *P* = 0.008, Figure 3). In the South region, the opposite was true. A higher state rural population percentage was associated with decreased orthopaedic surgeon density (adjusted R^2^ = 0.469, *P* < 0.01, Figure 4).

The state of Wyoming had the most orthopaedic surgeons per capita in the 2016 report. New Hampshire, Montana, Vermont, and Alaska had the next highest density of orthopaedic surgeons in 2016. This was similar to what was seen in the 2002 report (Table 4).

**Table 4:** Comparison of the Top Five States in Orthopaedic Surgeon Density From 2002 to 2016 and Their Overall Population Rank Among the 50 States and Washington D.C

2002			2016		
State	Orthopaedic Surgeon density	Population Rank	State	Orthopaedic Surgeon density	Population Rank
Montana	10.0	43	Wyoming	15.2	51
Wyoming	9.9	51	New Hampshire	14.7	41
Alaska	9.6	48	Montana	14.4	43
Washington, DC	9.6	49	Vermont	13.9	50
New Hampshire	9.4	41	Alaska	13.0	48

Orthopaedic surgeon density is reported as the number of orthopaedic surgeons per 100,000 residents. Data were obtained from the 2002 and 2016 Orthopaedic Practice in the United States reports published by the AAOS. Overall state population rank is based on US Census Bureau data from the corresponding year.

Texas had the lowest number of orthopaedic surgeons per capita in the 2016 report. Mississippi, Nevada, West Virginia, and Arkansas had the next lowest density of orthopaedic surgeons in 2016. This differs somewhat from what was seen in the 2002 report (Table 5).

**Table 5:** Comparison of the Bottom Five States in Orthopaedic Surgeon Density From 2002 to 2016 and Their Overall Population Rank Among the 50 States and Washington D.C

2002			2016		
State	Orthopaedic Surgeon Density	Population Rank	State	Orthopaedic Surgeon Density	Population Rank
Michigan	4.2	10	Texas	7.2	2
West Virginia	4.3	38	Mississippi	7.2	34
Mississippi	4.3	34	Nevada	7.3	32
Texas	5.1	2	West Virginia	7.5	38
New Mexico	5.2	36	Arkansas	7.5	33

Orthopaedic surgeon density is reported as the number of orthopaedic surgeons per 100,000 residents. Data were obtained from the 2002 and 2016 Orthopaedic Practice in the United States reports published by the AAOS. Overall state population rank is based on US Census Bureau data from the corresponding year.

## Discussion

Demand for arthroplasty procedures is expected to continually increase in the coming years,^1,4,5,21^ and there is a projected shortage of orthopaedic surgeons needed to meet this demand.^3,22^ Given these projections, evaluating local trends in TJA volume, including how and where TJA services are being administered, and the distribution of orthopaedic surgeons at the state level could help mitigate this shortage of orthopaedic surgeons. Our results demonstrate a decreased proportion of TJA procedures conducted in a rural setting over the study period and a slower growth rate of TJA procedure volume in rural hospitals as compared with urban hospitals. No notable change was observed in the proportion of TJA procedures conducted at teaching versus nonteaching hospitals in the urban setting. Linear regression analysis of orthopaedic surgeon density at a state level demonstrated a trend toward an increased number of orthopaedic surgeons per capita associated with decreased overall state population. In the South region, increased orthopaedic surgeon density was associated with increased state population density. A higher percentage of the population living in rural areas was associated with decreased orthopaedic surgeon density in the South and increased orthopaedic surgeon density in the rest of the country.

The finding that the proportion of TJA procedures conducted in rural hospitals decreased from 2000 to 2014 both regarding total volume and on a per capita basis could be related to multiple factors. First, this could be related to an increased willingness of patients living in rural areas to travel to high-volume centers in urban areas for TJA care. The fact that urban TJA volume demonstrated such a notable increase over the study period would support this possibility. This may ultimately be a positive development in rural TJA care. There is evidence that a decrease in the number of TJA procedures conducted at smaller, rural hospitals with lower overall volume and a subsequent increase in referral to larger, higher volume, urban centers may correlate with better patient outcomes. Higher volume hospitals have been shown to be associated with shortened length of stay and reduced early complication and 90-day readmission rates.^23-25^

Our results also correspond with previous studies which have demonstrated an overall decrease in the number of orthopaedic surgeons practicing in rural areas since the turn of the century.^9,26^ Although an increased proportion of TJA procedures being conducted in high-volume, urban centers may ultimately result in improved outcomes; as discussed earlier, future studies are needed to ensure that rural patients are indeed receiving appropriate TJA care and that this decreased proportion of TJA volume in rural areas is not because of a lack of access to providers. Should this be the case, it may be necessary to identify specific urban centers of excellence for complex primary and revision TJA procedures and ensure efficient referral pathways to these centers from underserved rural areas.

Regarding urban hospitals, our results showed no notable change in the proportion of TJA procedures conducted at teaching hospitals as compared with nonteaching hospitals, suggesting that both teaching and nonteaching hospitals will likely continue to see increased growth in TJA volume over the next several years.

Linear regression analysis showed an association between decreased state population and increased orthopaedic surgeon density, and this was reflected in the states that had the highest density of orthopaedic surgeons over the study period. In addition, linear regression analysis showed that states with a higher percentage of residents living in rural areas already have a relative abundance of orthopaedic surgeons as compared with more urban states with larger populations. Thus, on a national level, states with lower populations do not seem to have notable difficulty attracting a relative abundance of orthopaedic surgeons. This would seem to support the assertion that the decreased proportion of TJA volume in rural areas is related to rural patients seeking access to care in urban centers rather than a shortage of physicians because largely rural states actually had a relative abundance of orthopaedic surgeons.

For the South region, there was a notable correlation between decreased population density and decreased number of orthopaedic surgeons per capita. This suggests that highly rural states in the South have a relative shortage of surgeons as compared with those states with increased population density. This finding was confirmed with linear regression analysis of orthopaedic surgeon density and states' percentage of the population living in rural areas. For the South region, there was a negative association between the number of orthopaedic surgeons per capita and the rural population percentage, demonstrating that highly rural states in the South region have a relative scarcity of orthopaedic surgeons. Additional studies are needed to ensure that patients in the rural South have appropriate access to TJA care given the decreased proportion of TJA volume in rural areas seen in our study in conjunction with the relative scarcity of surgeons in rural Southern states.

This study has a number of limitations. The primary limitation is that this study involved the use of a large administrative database, which has inherent shortcomings. The accuracy of any data in these databases is limited to the accuracy of the codes that are entered into discharge records of the patients included in the database. The diagnoses that are input into the database are generally limited to those that are reimbursable, potentially limiting the diagnoses that are entered. Furthermore, the NIS database represents a stratified sample of discharge records, which we have used for the projection of national data. Although this is generally acceptable for large patient groups, the data included in the NIS may not provide an accurate representation of national procedural volume among minority subpopulations given the random inclusion of patients in the database. This limits the ability to make projections related to minority subsets. Despite this limitation, we think that our results represent the most accurate predictions available for TJA volume in the United States. This is especially true because we were primarily interested in evaluating trends in procedures conducted in urban teaching and nonteaching hospitals and rural hospitals, which does not require analysis of minority subset data. Given this and the limitations inherent to other available databases,^27^ we think that the NIS provides the most comprehensive data set for evaluating recent trends in TJA volume for the purposes of this study.

In addition, the AAOS Orthopaedic Practice in the United States reports represent the most comprehensive data set on orthopaedic surgeons in the United States to the best of our knowledge. However, these reports are not without limitations. The practice location used to determine orthopaedic surgeon density is based on registration with the AAOS and licensing boards.^7^ However, this registration does not allow for the selection of multiple practice locations, which makes it difficult to account for surgeons with atypical practice patterns such as locum surgeons or providers who practice in multiple states. Although it is not possible to account for locum surgeons who practice in multiple locations, locum surgeons account for a very small percentage of all orthopaedic surgeons (∼1%),^7^ so the ability to account for these surgeons in our analysis would likely not markedly change our results. It is not possible to determine the number of physicians who practice in multiple states. This is commonly seen in large urban areas that are close to state borders, such as the New York Tristate and Philadelphia areas. However, it can be argued that this limitation is largely an issue associated with urban areas and, if anything, would lead to an underestimation in the number of providers servicing urban regions. Rural region estimates would be less likely to be affected. Nevertheless, additional investigation into the distribution of locum surgeons and surgeons who practice in multiple states may provide more definitive clarity regarding our regional comparisons and projections.

## Conclusion

The proportion of TJA procedures conducted at rural hospitals has decreased since 2000. Urban teaching and nonteaching hospitals continued to see equivalent proportional growth over this period, which outpaced TJA volume growth of rural hospitals. In the South region, states with smaller, more rural populations had a relative scarcity of orthopaedic surgeons, indicating that decreased access to TJA care in rural areas in the South is potentially related to an inadequate supply of orthopaedic providers in these states. In the rest of the country, states with smaller, more rural populations had a higher density of orthopaedic surgeons. This may indicate an increased willingness of patients living in rural areas to travel to high-volume, urban centers for total joint care, which may lead to better overall outcomes. Future studies are needed to ensure that patients living in rural areas have appropriate access to TJA care.
